# A Frozen Plant DNA Language Model for Computational Prioritization and Functional Annotation of Melon (*Cucumis melo*) Variants

**DOI:** 10.3390/genes17101173

**Published:** 2026-09-23

**Authors:** Guosheng Sun, Yanping Wei, Entong Li, Mengting Xiao, Zhilin Zhang, Zhenchao Zhang, Zhongliang Dai, Zhihu Ma, Changwei Zhang

**Affiliations:** 1 Zhenjiang Institute of Agricultural Sciences in Hilly Area of Jiangsu Province, Zhenjiang 212400, China; sgs8725@jaas.ac.cn (G.S.);; 2 State Key Laboratory of Crop Genetics and Germplasm Enhancement, College of Horticulture, Nanjing Agricultural University, Nanjing 210095, China; 2021204020@stu.njau.edu.cn (E.L.); aa18451979903@163.com (Z.Z.); 3 Biological Breeding Laboratory, Xinjiang Academy of Agricultural Sciences, Urumqi 830091, China; weiyanping@xaas.ac.cn

**Keywords:** *Cucumis melo*, PlantCAD2, genomic foundation models, variant-effect prediction, cis-regulatory elements, model-guided breeding

## Abstract

**Background/Objectives:** Fruit quality in melon is determined by complex traits including sugar accumulation, aroma, flesh color, ripening behavior and disease resistance. Conventional genomic selection provides limited mechanistic interpretation, especially for regulatory and de novo variants. We adapted the EVEE interpretable embedding–probing framework to melon using PlantCAD2 as a frozen DNA language model backbone. **Methods:** The model was not fine-tuned or further pre-trained. Instead, lightweight probes were trained on fixed embeddings derived from sequence windows surrounding genetic variants. The pipeline integrated supervised variant-effect prediction, functional perturbation profiling, training-free masked log-likelihood ratio scoring, and cis-regulatory element generation. We applied the framework to 32,268 public genotyping-by-sequencing (GBS) SNPs from a melon diversity panel. A total of 30,052 variants were successfully encoded and analyzed. **Results:** Of these, 31.9% were classified as high predicted functional effect under the GWAS-proximal probe. We adopted a rigorous independent split based on chromosome and locus blocks to ensure test variants and training variants occupy distinct linkage-disequilibrium blocks. In this setting, the covariance probe yields an AUROC of approximately 0.51 with a 95% confidence interval covering 0.50, indicating no predictive advantage over random guessing. The full-cohort AUROC value of 0.748 reported earlier is attributed to linkage-disequilibrium leakage and overlapping positive samples between datasets, and thus does not reflect true independent generalization. The 17-class structural annotation probe reached 95.97% per-position accuracy. Functional perturbation signals were enriched in intronic, coding, and proximal promoter regions, consistent with known regulatory architectures in melon. Using the masked language model head with Gibbs sampling, we generated 76 candidate 400 bp cis-regulatory elements associated with sugar metabolism pathways. A posterior consistency check against 18 published GWAS/QTL loci showed effect-score patterns broadly concordant with reported loci; this is a consistency analysis against known loci, not an independent prospective validation. Variants in the *CmTST2* region exhibited elevated mean effect scores of 0.7922 compared with the genome-wide background of 0.406. **Conclusions:** Overall, this study provides a computational, hypothesis-generating framework in which a frozen plant DNA language model combined with lightweight probes prioritizes melon variants and generates candidate regulatory sequences; all outputs are unverified in-silico predictions requiring experimental validation before any functional or breeding use. Because every variant is scored independently from its local sequence context, the framework necessarily treats genetically linked, network-dependent traits as single-locus effects and does not model epistasis, pathway-level interactions, or genotype-by-environment effects; the outputs are sequence-level priors, not phenotype predictions. The results support a generalizable framework for model-guided functional genomics and precision breeding in Cucurbitaceae crops.

## 1. Introduction

Melon (*Cucumis melo* L.) is one of the most widely cultivated horticultural species in the Cucurbitaceae and is valued for the exceptional diversity of its fruit-quality traits, which include sweetness, aroma, flesh color, ripening behavior and shelf life [1,2]. Domesticated from a wild progenitor of south- or central-Asian origin, melon has been subjected to long-term selection that produced hundreds of cultivar groups spanning an unusually broad phenotypic spectrum, making it an attractive model for dissecting the genetic architecture of complex agronomic traits [3]. These cultivar groups are horticultural rather than formal taxonomic ranks. Current infraspecific classification recognizes two subspecies, ssp. *melo* and ssp. *agrestis*, within which the groups commonly cited in the breeding literature (for example Cantalupensis, Reticulatus, Inodorus, Conomon and Chinensis) are delimited by fruit morphology, use and horticultural history rather than by reproductive isolation; population-genomic data support the subspecies division and multiple independent domestication events, while showing that boundaries between groups are porous and that admixture between them is common [3,4]. Fruit sweetness, the single most commercially important trait, is determined chiefly by the accumulation of sucrose and other soluble solids, a process controlled by sugar-transporter genes, sucrose-cleaving invertase activity and sucrose synthase-mediated phloem unloading [5]. The characteristic melon aroma arises from the biosynthesis of volatile esters through the concerted action of lipoxygenase cascades, alcohol dehydrogenases and acyltransferases acting on fatty-acid-derived substrates [6]. The extent of experimental support differs markedly between the steps of this pathway in melon itself. The alcohol acyltransferase family has been functionally characterized in melon, including enzymatic assays on recombinant CmAAT proteins that identified a threonine residue essential for activity [6], whereas the contributions of the upstream lipoxygenase cascade and of the alcohol dehydrogenases have in melon been inferred largely from expression profiling and biochemical correlation rather than from transgenic or knockout validation, so their causal roles in this species remain to be confirmed. Flesh pigmentation depends on carotenoid biosynthesis and is governed by master regulatory loci such as *CmOr*; ripening, by contrast, is tightly coupled to ethylene signaling in climacteric germplasm but proceeds independently of ethylene in non-climacteric cultivars [7,8]. The applied relevance of *CmOr* is well established: the causal ‘golden’ SNP is diagnostic for orange flesh across diverse germplasm and has been converted into a co-dominant marker that is used directly in marker-assisted selection for flesh color, which illustrates how a single well-resolved allelic variant can pass from mapping into routine breeding practice [9]. Disease resistance likewise has a tractable genetic basis amenable to molecular dissection [10]. Together, these traits constitute prime targets for genomics-driven breeding improvement.

The successive release of high-quality reference genomes, pan-genomes and telomere-to-telomere (T2T) assemblies has fundamentally rescaled the catalog of genetic variation accessible in melon. The first chromosome-level reference enabled population-scale discovery of SNPs and InDels, and connected sequence polymorphisms to QTL for sugar content, disease resistance and sex determination [3,11]. Subsequent pan-genome analyses revealed that structural variation, including presence/absence variation and copy-number variation, accounts for a substantial fraction of the phenotypic diversity in flesh color and sugar accumulation that escapes detection by SNP arrays alone [12,13]. More recently, T2T assemblies have resolved centromeric and pericentromeric repeats that harbor functionally important regulatory sequences, providing an unprecedented basis for comprehensive variant annotation [14]. Resequencing of hundreds of diverse accessions has further enabled GWAS and selective-sweep analyses that have recorded genomic footprints of domestication and improvement [3,4]. Despite this progress, mechanistic interpretation of non-coding and regulatory variation remains a critical bottleneck: most GWAS-significant signals localize to intergenic or intronic regions, where identifying causal variants requires functional evidence beyond statistical association [15].

Genomic prediction models, in particular genomic best linear unbiased prediction (GBLUP) and ridge-regression BLUP (rrBLUP), have become standard tools for estimating breeding values from genome-wide marker data in plant species [16,17]. When the training and target populations share similar genetic backgrounds, these models effectively capture polygenic effects and have proved useful for predicting complex traits, including yield, disease resistance and fruit quality, in cucurbits [18,19]. GBLUP and rrBLUP nonetheless implicitly assume that all markers contribute approximately equally to genetic variance, which prevents them from explaining mechanistically how coding, splice-site, or cis-regulatory variants drive phenotypic differences [20]. This equal-weighting assumption is especially restrictive for non-coding regulatory variation. Marker effects are estimated from linkage disequilibrium with unobserved causal sites rather than from the regulatory consequence of the sequence itself, so promoter, enhancer, untranslated-region and splice-region variants are captured only indirectly; their effects are neither spatially localized nor mechanistically resolved, and they cannot be transferred to a population in which the marker causal–variant phase differs [15,20]. For quality traits that are heavily influenced by regulatory variation, such as sugar loading, ester-based aroma biosynthesis and the initiation of ripening, statistical prediction alone is insufficient to identify causal variants, motivating an integrated framework that combines functional annotation with variant-effect prediction [15,21,22]. Stated plainly, the limiting property of these statistical predictors is not their accuracy but their lack of mechanistic interpretability: they return a breeding value without indicating which sequence feature is responsible for it, and it is precisely this gap that sequence-based foundation models are intended to fill.

Large-scale DNA foundation models have opened an alternative route to sequence-based functional annotation that does not depend on extensive experimental labels. By self-supervised pre-training on cross-species genomic sequences, these models learn evolutionary constraint, regulatory grammar, and long-range dependencies, and transfer the resulting representations to downstream tasks such as regulatory-element annotation, expression prediction, and variant prioritization [23,24]. In plants, AgroNT, trained on 48 reference genomes, has shown practical utility for predicting promoter and terminator strength, inferring tissue-specific expression and screening functional variants [25]. PlantCaduceus subsequently enabled single-nucleotide-resolution modeling across multiple angiosperm genomes while explicitly accommodating the reverse-complement symmetry of double-stranded DNA [26]. PlantCAD2 extends this framework to longer sequence context and a broader angiosperm phylogeny, improving cross-species regulatory annotation [27]. PlantRNA-FM has further demonstrated that the foundation-model paradigm can be extended to the RNA level by integrating sequence and secondary-structure information for plant-specific transcript modeling [28]. RNA-level and DNA-level models are complementary rather than competing. A DNA model scores the consequence of a variant in its genomic context and can therefore reach promoter, enhancer and intronic sequence that is never transcribed into mature mRNA, whereas an RNA model captures how the resulting transcript is spliced, folded, stabilized and translated. Coupling the two in a crop setting allows a candidate regulatory or splice-region variant to be traced from genomic sequence through transcript fate to a proposed molecular mechanism, which neither layer can achieve alone [28,29]. Together, these efforts establish the feasibility of learning generalizable sequence representations that serve plant functional genomics [29]. Important limitations nonetheless persist. Systematic benchmarking has shown that predictive performance varies markedly with architecture, tokenization strategy, and pooling scheme, and that pre-trained representations do not always outperform conventional k-mer or one-hot baselines on certain regulatory tasks [30]. The magnitude of this task dependence is illustrated quantitatively by the present study, in which one frozen backbone yields 95.97% per-position accuracy on structural annotation but an AUROC of only approximately 0.51 on supervised variant-effect prediction under linkage-disequilibrium-independent splits. The same embeddings therefore range from near-saturated to entirely uninformative depending on the task and on the provenance of their labels, which is the practical form that benchmark variability takes in a non-model crop. This suggests that current plant foundation models do not yet fully capture the complexity of plant regulatory grammar, particularly in lineages with high repeat content or limited training data [29]. Furthermore, the adaptation of human-centric variant effect–prediction frameworks—such as EVEE (enforced variant effect estimation), which interprets sequence perturbations using frozen-model embeddings and lightweight probes—to crop species remains largely unexplored [24,31]. The heterogeneity of plant genomes, the relative scarcity of experimentally validated causal variants, and the computational cost of full-parameter fine-tuning together constitute substantial barriers to deploying these methods directly within crop breeding pipelines [29,32]. Label scarcity is the binding constraint in this setting, and it is most acute in non-model horticultural crops such as melon, for which no curated catalog of experimentally validated causal variants exists. This is precisely the regime in which a hypothesis-generation framework built on frozen embeddings and lightweight probes, rather than a supervised predictor that presupposes abundant gold-standard labels, is the appropriate design choice.

Several knowledge gaps are particularly conspicuous in melon. Experimentally validated causal variants remain scarce, the regulatory architecture of key quality-trait genes is incompletely characterized, and structural variation is not yet integrated systematically with sequence-based functional annotation [33,34]. These limitations make it difficult to construct genuinely independent labels for supervised variant-effect prediction. Here, we evaluate a PlantCAD2–EVEE-inspired workflow that extracts contextual embeddings from a frozen PlantCAD2 backbone and applies lightweight probes to produce GWAS-proximal functional-prior scores for variants in coding regions, splice sites, promoters, and candidate cis-regulatory elements. The supervised output is treated as a population- and label-specific ranking, not as a causal-effect or deleteriousness estimate. We emphasize that GWAS-proximal labels are noisy positional proxies and not causal annotations. A variant is labeled positive because it lies within 20 kb of an associated marker, so in a species with extended linkage disequilibrium the positive class necessarily absorbs many non-causal neighbors, while genuinely causal variants at loci that did not reach genome-wide significance are assigned to the background. Both directions of error are systematic rather than random, and neither can be corrected by rebalancing or regularization. Complementing this, training-free masked language–model likelihood scoring provides an annotation-free prioritization layer for genomic regions that lack experimental labels [33]. High-effect variants are mapped to genes implicated in sugar accumulation, volatile-ester biosynthesis, carotenoid pigmentation, and ethylene-dependent ripening. For prioritized regulatory candidates, model-guided in silico mutagenesis generates experimentally testable hypotheses about causal sequence features, rather than equating computational predictions with experimentally validated regulatory designs [34]. Any high-effect variant or designed element nominated by this workflow consequently requires experimental confirmation before it can support a functional claim: at minimum reporter or dual-luciferase assays together with allele-specific expression for regulatory candidates, and transient or stable transformation with phenotypic assessment for the strongest candidates [21,34]. To assess predictive reliability, we performed external consistency validation of candidate-gene effect predictions and chromosome-level deleterious-enrichment rankings against 18 previously reported GWAS/QTL-significant loci [9,35,36]. Because simple sequence, annotation, and pooled-embedding baselines were not available under the same blocked splits, the study does not claim that PlantCAD2 improves predictive performance over simpler alternatives. Its primary contribution is a transparent hypothesis-generation workflow and an empirical assessment of the limits of GWAS-proximity supervision in crop variant prioritization. We state at the outset that melon fruit-quality traits are emergent properties of multi-gene regulatory and metabolic networks operating under particular developmental and environmental conditions. No framework that operates on sequence context alone, including the one evaluated here, can therefore be expected to account fully for phenotype, and the per-variant scores reported below are locus-level priors rather than phenotype predictions.

## 2. Materials and Methods

### 2.1. Data Sources and Preprocessing

The melonv4.0 assembly of the doubled-haploid line DHL92 served as the reference genome; the soft-masked FASTA and GFF3 annotation were obtained from Ensembl Plants release 62 [11,37,38]. Population variant calls were taken from the GBS SNP VCF distributed by the Cucurbit Genomics Database [39,40] and were filtered to minor-allele frequency (MAF) ≥ 0.01 and missingness ≤ 0.5. Chromosome identifiers chr01–chr12 in the VCF were mapped to Ensembl contig labels using a fixed alignment; a short contig containing only 27 variants was retained but excluded from chromosome-level interpretation. The analyses reported here use the GBS SNP dataset; although the interface also accepts short InDels, all in-text statistics refer to GBS SNPs.

Supervised labels were derived from a companion table of GWAS-significant SNPs for melon fruit-quality traits: variants within 20 kb upstream or downstream of any significant SNP were marked positive as GWAS-proximal candidates, and all others were treated as background. This proximity-based labeling scheme is subject to linkage-disequilibrium confounding: many linked non-causal variants are mislabelled as positive, representing a major source of label noise that constrains supervised performance. Because melon lacks a catalog of experimentally validated causal variants, this label scheme is defined by physical proximity rather than functional verification; we therefore refer to it as a GWAS-proximal label throughout and discuss the resulting noise when interpreting probe performance. The diversified regions and selective-sweep intervals reported in the melon population variation map [3] and the 297-accession resequencing study [4] were used as complementary domestication- and improvement-related annotations for biological interpretation.

### 2.2. PlantCAD2 Sequence Encoding and Embedding Extraction

PlantCAD2-Small-l24-d0768 was used as a frozen DNA encoder [26,27]; it implemented a Caduceus-based, bidirectional reverse-complement-equivariant architecture and was pre-trained with a masked-language-modeling objective on angiosperm genomes [26,27,41]. Weights and tokenizer were loaded through the Hugging Face AutoModel interface with trust_remote_code = True [42]; all parameters were kept frozen, and no fine-tuning was performed.

For each variant, reference- and alternative-allele window sequences no longer than PlantCAD2’s context limit of 8192 bp and centered on the variant site were extracted from the DHL92 reference genome. The reference and alternative sequences were separately passed through the frozen PlantCAD2 encoder, and final-layer hidden states were extracted. The model hidden states had shape [L, 2 × 768], where the first 768 channels represented the sense strand and the second 768 channels represented the reverse-complement strand; the latter was flipped along the length axis and mapped back to sense-strand coordinates. Per-position cosine divergence between the reference and alternative alleles was then computed in the shared coordinate frame; the K positions with the largest divergence were selected (default K = 256), and the hidden vectors from the two strands, two alleles, and Top-K positions were stacked into a [2,2,K,768] activation tensor (strand × allele × Top-K position × hidden dimension), matching the EVEE input layout [31]. All activation tensors were precomputed with the frozen model and cached to disk in safetensors format for reuse in downstream probe training and inference; variants that triggered CUDA or operator-kernel exceptions during encoding were recorded and skipped by the exception-handling mechanism, allowing the batch pipeline to continue running.

### 2.3. Variant-Effect Prediction

The covariance probe was adapted from EVEE [31], with the input dimension set to 768 to match PlantCAD2-Small. For each strand, non-shared linear maps projected the alternative-minus-reference embedding difference from 768 to 64 dimensions. Cross-covariances were computed over the Top-K positions, summed across strands, diagonally regularized, and square-root compressed using Newton–Schulz iterations to reduce dominant eigenvalue effects. The compressed covariance was then passed through a factorized bilinear readout, and the two output logits were converted by softmax into a deleteriousness probability. The probe was trained with cross-entropy loss and AdamW [43] using a learning rate of 1 × 10^−3^, weight decay of 1 × 10^−4^, batch size of 32, and 30 epochs. The 6022 labeled variants were split into 4818 training and 1204 validation variants; training negatives were downsampled to an approximately 1:2 positive:negative ratio, and validation AUROC was used for model selection. For training-free masked log-likelihood ratio (LLR) scoring, each SNV position was replaced with [MASK], and PlantCAD2 predicted the nucleotide distribution at that site. The score was computed as LLR = log P(alt | context) − log P(ref | context). Negative LLR values indicate that the alternative allele is less compatible with the model-learned evolutionary constraints, providing label-independent supporting evidence.

### 2.4. Functional Annotation and Perturbation-Profile Analysis

The per-position annotation probe followed EVEE’s annotation–perturbation design [31]. It used the reverse complement (RC)–invariant embedding as input, obtained by taking the per-position mean of the sense- and reverse-complement-strand representations, and applied a linear layer to map the 768-dimensional hidden vector to multi-label functional annotation probabilities. We first defined an annotation panel containing 34 melon functional categories, covering region identity, splicing, codon position, promoter/terminator features, transcription-factor binding, chromatin accessibility, and melon-related pathways including sugar accumulation, aroma, ripening, carotenoid flesh color and sex determination. Because transcription-factor binding sites and chromatin-accessibility categories require experimental data such as DAP-seq or ATAC-seq, the present analysis trained only the 17 structural annotations directly derivable from the DHL92 GFF3 annotation [11,38], including CDS, intron, 5′/3′ UTR, intergenic region, exon boundary, splice donor/acceptor, codon positions 1–3, start/stop codons, frameshift risk, core/proximal promoter and terminator. The probe was trained for 20 epochs on 237,568 labeled positions using weighted binary cross-entropy loss and produced 17-category multi-label probabilities.

To obtain variant-level functional perturbation profiles, the reference- and alternative-allele window embeddings were passed separately through the annotation probe and converted to category probabilities by sigmoid activation. For each variant, the signed difference between alternative and reference probabilities was computed within a ±5 bp window around the variant site. For each category, the value with the largest absolute magnitude in this window was taken as the representative perturbation. Categories were then ranked by absolute perturbation to produce the local functional perturbation profile for each variant.

### 2.5. Genome-Wide Inference, Sequence Design and Breeding-Relevant Evaluation

Genome-wide inference integrated the outputs of the covariance probe, masked LLR and annotation probe. The covariance probe assigned a predicted functional-effect probability to each scorable variant, using 0.5 as the binary classification threshold; masked LLR provided label-independent evidence of sequence constraint; and the annotation probe produced local functional perturbation profiles. The three outputs were merged by variant coordinate into a unified results table. To assess chromosome-level deleterious burden, contig coordinates were first converted to chromosome identifiers using the predefined mapping, after which the number of scorable variants, number of deleterious variants, summed deleteriousness probability and mean effect score were calculated for each chromosome. The short unplaced contig was retained in the global results table but excluded from chromosome-priority interpretation.

De novo regulatory elements were designed using the PlantCAD2 masked language–model head through iterative Gibbs masked resampling. Each candidate sequence was initialized from a random DNA sequence; in each iteration, a subset of positions was randomly masked, PlantCAD2 predicted the nucleotide distribution at the masked sites, and bases were resampled from {A,C,G,T} at a temperature of 1.0. Designs were anchored on sugar-accumulation pathway genes, including *CmTST2*, *CmSPS* and *CmAGA2*, to generate 400-bp candidate regulatory sequences. All designed sequences were treated as computational candidates and require further probe/LLR screening and experimental validation, such as reporter assays, allele-specific expression, RT–qPCR, transient expression or stable transformation, before functional or breeding use can be inferred.

All components were implemented in PyTorch (v2.12.1) [44] and used Transformers (v5.10.4) [42], mamba-ssm (v2.3.2) and causal-conv1d (v1.6.2) for PlantCAD2 inference. Genome-wide analysis was run on a single NVIDIA L20 GPU (48 GB, CUDA 11.8; NVIDIA Corporation, Santa Clara, CA, USA). A lightweight CPU mock encoder was also implemented for continuous integration and smoke testing. Classification performance was evaluated using AUROC, AUPRC, F1 score and confusion matrices, whereas regression-mode evaluation used MSE and Pearson r. Sample size was naturally determined by the number of GBS SNPs for which coordinate harmonization, window extraction and activation caching could be completed. The analysis pipeline (melon-plantcad2-evee-v1.0), probe weights, prediction tables, chromosome-burden summaries, designed-element FASTA files and mock encoder are provided as https://github.com/sgs8725/melon-plantcad2-evee-v1.0 (accessed on 21 September 2026).

No new plant materials were generated, and no field or wet-laboratory experiments were performed in this study. Therefore, all variant rankings, chromosome priorities and de novo designed elements should be regarded as computational hypotheses rather than experimentally validated functional conclusions.

### 2.6. Model Validation

To evaluate the ability of the model to discriminate effects at known functional loci, we designed two complementary validation schemes. For Scheme A, termed candidate-gene coordinate validation, we collected 18 previously reported melon GWAS and QTL significant loci spanning flesh color at *CmOr* [9], soluble solids content at SSC QTL [35], sugar transport at *CmTST2* [45], fruit shape at *CmACS7* [36], aroma at *CmAAT* [4], and bitterness at *CmBt* [3]. For each candidate gene, we counted the GBS SNPs within a ±100-kb genomic coordinate window, computed the mean predicted effect score and the proportion of high-effect variants with scores greater than or equal to 0.7, and compared these values with the genome-wide background mean of 0.406. In regions with insufficient GBS coverage, where no variants were present within the window, the effect score of the nearest variant was used as a neighborhood reference. For Scheme B, termed chromosome-level deleterious-enrichment validation, we compared the per-chromosome fraction of deleterious variants with previously reported domestication-selection signals and selective-sweep intervals [3,4], thereby testing whether the chromosomes nominated by the model as having high burden agreed with documented domestication and improvement hotspots. Both schemes compare model predictions against candidate genes and genomic intervals drawn from the published literature; while these loci were not explicitly included in the GWAS-proximal training label construction, they may share underlying trait signals and population structure with the training data, so this constitutes a posterior consistency check rather than a prospective independent validation under randomized controls defined by GWAS-proximal windows, serving as external references to assess the biological plausibility of the predicted effects.

## 3. Results

### 3.1. End-to-End Pipeline from Population VCF to Interpretable Predictions

The melon variant interpretation system comprises three tiers: a frozen PlantCAD2 foundation model layer, a lightweight trainable/parameter-free probe layer, and a breeding decision and sequence-design layer (Figure 1). The third tier is conceptual in the present study. It specifies the interface through which ranked variants and designed sequences would be handed to a breeding program, but no marker derived from these scores was deployed, no cross was made, and no designed element was synthesized or assayed here; its practical downstream application therefore remains to be demonstrated and is stated as an intended use rather than a realized one. Analysis begins with melon GBS VCF data, the DHL92 reference genome (melon v4.0), and GFF3 annotation. The dataset builder generates 32,268 reference/alternative allele sequence windows centered on each variant locus, spanning up to the PlantCAD2 context hard limit of 8192 bp. The frozen PlantCAD2-Small model with 88 M parameters performs a single forward pass per variant—encoding reference and alternative alleles independently—and simultaneously obtains per-position hidden states for both sense and antisense strands via reverse-complement equivariance. Positions exhibiting the greatest divergence (top K = 256 by cosine divergence) are selected and stacked into a [2,2,K,768] activation tensor (strand × allele × Top-K position × hidden dimension).

During genome-wide batch encoding, PlantCAD2 successfully cached 30,052 activation tensors; 2215 variants were flagged and skipped owing to causal-conv1d CUDA kernel exceptions captured by an error-handling routine. These failures originate in the causal-conv1d kernel rather than in any property of the variants themselves, so they would be expected to be independent of chromosome and of variant class. Empirically, however, this expectation is violated: a χ^2^ test of skipped versus successfully encoded variant counts across the 13 genome assemblies rejects homogeneity (χ^2^ = 6345.9, df = 12, *p* < 0.001), with the skipped variants confined to four chromosomes (chr11: 868; chr06: 537; chr08: 466; chr04: 345 variant records) and no skips on the remaining eight chromosomes or the unanchored contig. Both the skipped and the successfully encoded sets consist exclusively of SNVs, so no variant-class contrast is possible. The concentration therefore reflects a systematic, assembly-dependent property of the kernel failure rather than a random loss. Because the affected chromosomes are retained in all downstream analyses and the skipped fraction is small (2215 of 32,267 variants, 6.9%), the loss does not change the ranking of chromosomes, but the per-chromosome counts in the table belowshould be read with this asymmetry in mind. By combining covariance probes, annotation probes, and a training-free masked LLR, the pipeline generated complete effect scores, binary class predictions, and functional disruption spectra for 30,052 variants (the counts 30,052/2215 are unified throughout this manuscript). Because the frozen backbone remains unchanged, all variant embeddings are precomputed once and cached as safetensors, enabling downstream probes to operate without re-running the backbone—thereby achieving genome-scale interpretable predictions at low computational cost.

A frozen reverse-complement-equivariant DNA foundation model encodes reference and alternative sequence windows into structured representations, which are processed by three downstream probes for variant effect prediction, functional annotation, and masked language model (MLM) scoring. These outputs are integrated to produce unified variant-level scores and downstream interpretations for large-scale genomic analysis.

### 3.2. Frozen PlantCAD2 Embeddings Enable Linear Decoding of Functional Genomic Information via Lightweight Probes

The 17-class structural annotation probe was trained on 237,568 genome-wide per-position labels derived from the DHL92 GFF3 annotation. The probe maps PlantCAD2’s reverse complement–invariant embeddings (per-position mean of sense and antisense strands) from 768 dimensions to a 17-dimensional multi-label annotation probability through a single linear layer. Per-position accuracy increased steadily from 94.60% at epoch 1 to 95.97% at epoch 20, while training loss fell from approximately 0.50 to 0.20 (Figure 2A). These results demonstrate that PlantCAD2 frozen embeddings linearly encode functional genomic information—including regional identity, splicing context, and codon frame—providing a foundation for downstream disruption spectrum analysis. The 17 classes are substantially imbalanced in site count, with introns and intergenic regions being the dominant categories; per-position accuracy is therefore biased toward majority classes, and probe performance on rare classes such as splice donor/acceptor sites and start/stop codons should be assessed together with per-class precision–recall metrics. Class imbalance was addressed during training rather than ignored: the annotation probe was optimized with a weighted binary cross-entropy loss in which each of the 17 categories contributes inversely to its site frequency, so that rare categories are not simply absorbed by the dominant ones. It must nevertheless be stated explicitly that the headline figure of 95.97% is a micro-averaged per-position accuracy and is therefore inflated by the majority classes; it is not uniform across the 17 categories and must not be read as the accuracy with which splice donor and acceptor sites or start and stop codons are recovered. We have now quantified this directly: the probe was re-trained with an identical pipeline on 24 of the 29 sampled windows (196,608 positions after balancing) and evaluated on the five held-out windows (40,960 positions), and precision, recall, F1 and AUPRC were then computed separately for each of the 17 annotation categories, together with the number of positive positions and the positive prevalence of each category in the held-out set, and summarized by a macro-averaged F1 and a micro-averaged per-position accuracy. Because what that evaluation establishes is the contrast between the dominant and the rare categories rather than the value attached to any individual category, we report it in the text that follows rather than as a separate table. The held-out micro-averaged per-position accuracy is 94.4%, consistent with the headline figure, but the class-wise view confirms the caution above. Performance is carried by the dominant categories—region_CDS (precision 0.62, recall 0.34, F1 0.44, AUPRC 0.57 against a positive prevalence of 0.067) and region_intron (recall 0.84 at precision 0.21)—whereas the biologically critical rare categories are largely not recovered on held-out sequence: splice_donor reaches precision 1.00 but recall 0.03; splice_acceptor, region_5UTR, region_3UTR, region_exon_boundary, promoter_core, terminator, start_codon and stop_codon are not recovered at all (F1 = 0, AUPRC at or near class prevalence); and codon-frame positions have recall below 0.01. Two categories (region_intergenic and frameshift_risk) contained no positive positions in the held-out windows. We therefore do not claim single-position recovery of splice sites, codon framing, or start and stop codons from frozen embeddings, and the disruption spectra reported in this study should be interpreted as region-level (CDS/intron/promoter-proximal) rather than fine-grained structural calls. The supervised covariance probe was trained using GWAS-proximal labels: variants within 20 kb of a GWAS-significant SNP were designated positive, and all remaining variants served as background. A total of 6022 labeled variants were split 4818/1204 into training and validation sets; negative samples were downsampled to a positive:negative ratio of approximately 1:2 to stabilize optimization. Downsampling rather than loss re-weighting was used for this probe, so the validation metrics reported below refer to a class-rebalanced set and are not directly transferable to the natural, strongly imbalanced genome-wide distribution; this is one reason why the class-balanced hold-out AUROC and the full-cohort AUROC are not comparable quantities. Training used an AdamW optimizer (learning rate 1 × 10^−3^, weight decay 1 × 10^−4^, batch size 32) for 30 epochs. On the class-balanced hold-out validation set (*n* = 1204), validation AUROC peaked at 0.5878 at epoch 28 (Figure 2B; Table 1). Under rigorous chromosome-block (locus-block) independent evaluation—in which no test variant shares an LD block with any training variant—the probe attains an AUROC of approximately 0.51 (95% CI includes 0.50), statistically indistinguishable from random chance. The monotonic decrease from 0.748 (full-cohort, training-positive overlap) → 0.5878 (class-balanced random hold-out with within-chromosome LD) → 0.51 (chromosome-block independent) is the signature of linkage-disequilibrium leakage rather than true generalization. For readers less familiar with population genetics, this form of leakage arises as follows. Alleles at physically proximate loci are co-inherited and therefore statistically correlated within a population; if a positive variant is placed in the training set while variants correlated with it fall in the test set, the probe can recover the test label from the shared haplotype background instead of from any functional property of the sequence. Because our positives are defined by physical proximity to association peaks, they occur in dense clusters, and leakage of this kind is severe unless whole chromosomes or whole linkage-disequilibrium blocks are held out. The monotonic fall in AUROC as the split becomes stricter is the direct signature of that effect. We therefore report the independent AUROC as the honest estimate and interpret all variant scores as hypothesis-generating priors constrained by the GWAS-proximal label noise. No score reported in this study should be interpreted as a causal prediction or as an estimate of effect size. The scores are prioritization signals, and their only defensible use is to order candidates for subsequent experimental testing. The full-cohort AUROC of 0.748 reflects non-independent evaluation (partial overlap with training positives via GWAS-proximity labeling) and is retained here solely as a within-population ranking metric, not as evidence of generalization. This genome-wide AUROC remains substantially below the approximately 0.97 AUROC reported in human ClinVar settings, primarily reflecting inherent noise in GWAS-proximal labels arising from linkage disequilibrium entrainment, small-effect contributions, and spurious associations, rather than a fundamental limitation of the frozen PlantCAD2 backbone.

### 3.3. Genome-Wide Landscape of Predicted Variant Effects

Predicted deleteriousness scores for all 30,052 scored variants span the full [0, 1] range, with a mean of 0.406 and a median of 0.397; the 25th and 75th percentiles are 0.023 and 0.694, respectively (Figure 3). The interquartile range is therefore 0.671, so the middle half of the distribution spans roughly two-thirds of the full score range, confirming that dispersion is extreme rather than concentrated about the mean. We report the interquartile range in preference to a standard deviation because a standard deviation is not an informative summary for a distribution of this shape: a strongly bimodal variable can return a moderate standard deviation while carrying almost no probability mass near its own mean. When applying a decision threshold of 0.5, 9596 variants corresponding to 31.9% of the dataset were categorized as deleterious, whereas 20,456 variants, making up 68.1%, were deemed neutral or benign. The value of 0.5 is simply the default decision boundary of the two-class softmax output and was fixed a priori; it was not tuned on validation data and no alternative cut-off was optimized, because tuning a threshold against labels that are themselves noisy positional proxies would manufacture apparent performance rather than measure it. To confirm that the binary call is a reporting convenience rather than the information-bearing output, we re-scored the 30,052 saved predictions at cut-offs of 0.4, 0.5, 0.6 and 0.7. The genome-wide high-effect fractions are 35.3%, 31.9%, 28.4% and 24.8%, respectively, and the per-chromosome ranking of high-effect fractions is stable across these cut-offs (pairwise Spearman rho = 0.70–0.97, median 0.88), confirming that the continuous score carries the signal and the threshold merely partitions it. Given the near-chance independent AUROC, the binary call should in any case be regarded as a reporting convenience; the rank order of the scores, rather than the two classes it induces, is the output we intend readers to use. The score distribution exhibits a pronounced bimodal pattern: a large cluster near 0 reflects variants confidently classified as neutral, a distinct cluster near 1 reflects variants confidently classified as deleterious, and a mode of ambiguous evidence sits near the median between the two poles. This bimodal pattern may reflect the label structure (GWAS-proximal positives vs. background) rather than intrinsic variant-effect distributions; a minority of high-scoring variants are embedded in a large low-scoring background, indicating that the probe imposes a reproducible ordering within this population, but not that the ordering generalizes. Because the bimodality is at least partly an artifact of how the labels were constructed, it cannot be taken as evidence about the true distribution of functional effects in the melon genome, and it places a hard limit on causal inference: the two modes separate variants that resemble the positive label set from those that do not, which is a statement about label geometry rather than about biology. The scores are therefore positioned strictly as prioritization tools.

Variants are classified using a 0.5 decision threshold into benign/neutral (68.1%) and deleterious (31.9%). The score distribution is bimodal, with median (0.397) and mean (0.406) located in the intermediate range. This shape is what makes genome-scale prioritization tractable despite label noise: a small, well-separated high-scoring tail can be taken forward for experimental testing at realistic cost, while the large low-scoring mass is de-prioritized. The practical value of the output therefore lies in the ordering it imposes on 30,052 variants rather than in the calibration of any individual score.

### 3.4. Disruption Spectra Reveal Introns, Coding Sequences, and Proximal Promoters as Predominant Disruption Categories

Functional disruption spectra from the annotation probe reveal a consistent hierarchy of disrupted annotation classes (Figure 4). Intronic context is disrupted by nearly every variant (99.3%), reflecting the dominant genomic footprint of introns. This near-saturation is a compositional and computational effect rather than a biological finding. Introns occupy the largest annotated share of the genome, so the plus or minus 5 bp window around a randomly sampled SNP is more likely to overlap intronic context than any other category, and the maximum-absolute-perturbation rule then almost always registers a non-zero intronic signal. The frequency of a category in this spectrum consequently reflects its genomic footprint combined with the sensitivity of the probe, and must not be read as a ranking of functional importance. CDS are implicated in 76.8% of variants, and proximal promoter context in 75.9%, a noteworthy signal suggesting that a substantial proportion of segregating variants fall within, or are modeled as affecting, regulatory regions, consistent with the biology of *Cucumis melo* quality traits being driven by cis-regulatory elements. The disruption frequencies of 58.9% for the 3′ UTR and 45.2% for the terminator are intermediate. Splice donor and splice acceptor sites are detected at high frequencies of 23.0% and 22.8%, respectively, relative to their limited genomic footprint, indicating that PlantCAD2 embeddings are sensitive to splicing signals. These splice signals were inferred entirely from the DHL92 GFF3 annotation. No experimental splicing evidence, such as melon Iso-Seq, junction-level RNA-seq, or minigene reporter assays, was used either to train the probe or to verify the detected perturbations, so the detections demonstrate sensitivity to annotated splice-site sequence grammar rather than confirmed alteration of splicing. Categories defined by narrow windows immediately flanking variant positions, including the intergenic region at 22.8%, the exon boundary at 22.8%, and codon position 2 at 15.4%, show lower detection rates, consistent with the computational strategy of taking the absolute maximum of local perturbation within each window, which renders detection of such narrow-window classes conservative. For breeding practice, the operational reading of this spectrum is the promoter signal rather than the intronic one. Proximal-promoter perturbation combines a high detection frequency with a comparatively small genomic footprint and with prior evidence that melon quality traits are driven by cis-regulatory variation, which makes promoter-proximal high-scoring variants, and in particular those upstream of sugar-metabolism and aroma genes, the most efficient first tranche for reporter-assay and allele-specific-expression validation. Intronic hits, by contrast, should not be prioritized on the strength of their frequency alone.

Percentages indicate variants disrupting each of the ten most frequently affected annotation classes. Colors denote coding/splicing, regulatory and non-coding/structural categories. The spectrum should be read against genomic footprint rather than as a ranking of biological importance: the near-universal intronic signal is a footprint effect, whereas detection of proximal-promoter, splice-donor and splice-acceptor perturbation at frequencies far above the genomic share of these features is the biologically informative result, indicating that the frozen embeddings retain regulatory and splicing grammar of direct relevance to melon quality traits.

### 3.5. Chromosomal Variant-Effect Burden as Computational Breeding Priorities

Mapping predictions onto individual chromosomes reveals substantial inter-chromosomal variation in predicted-effect burden (Figure 5; Table 2). Two complementary summary metrics provide informative, divergent rankings. By the mean per-variant effect score, which represents the predicted-effect enrichment per locus, chromosome 9 at 0.4442 and chromosome 1 at 0.4436 rank jointly highest, with equal high-effect variant proportions of 34.9%, jointly nominating these as the chromosomes most enriched for predicted high-effect alleles per locus. This enrichment is concordant with previously reported genetic architecture. Chromosome 1 is the chromosome most strongly enriched for domestication-selection signals in melon population-genomic studies, and chromosome 9 carries both the flesh-color locus *CmOr* and the bitterness locus *CmBt*, each a documented target of strong artificial selection [3,4,9]. This agreement is examined formally as Strategy B belowand is one reason for treating the chromosome-level ranking as more robust than any individual variant score. By the cumulative effect burden, defined as the sum of all variant effect scores on a given chromosome and equivalent to the scored variant count multiplied by the mean effect score, chr05 at approximately 1820 and chr01 at approximately 1722 lead. The lead of chr05 is primarily driven by carrying the largest number of scored variants, totaling 4443, rather than by an elevated per-variant effect, as it has an intermediate rank with a mean effect of 0.4096.

This divergence reflects two complementary breeding priority logics. Cumulative burden reflects the total count of high-scoring variants per chromosome. The agronomic favourability of these variants cannot be determined without phenotypic validation. This metric is suitable for generating hypotheses and setting priorities in marker-assisted selection; mean effect reflects the expected impact intensity per variant, more appropriate for causal-variant screening and fine mapping. Consistently, all five highest-impact variants reaching an effect score of 1.0000 are located on chr01, with predominant disruption categories concentrated in proximal promoter, intronic, and coding regions (Table 3), providing specific support for this chromosome’s priority. All five lie within the first 5 Mb of chromosome 1 and may therefore reside within one or a small number of linkage-disequilibrium blocks, as noted in the footnote to Table 3; all five are dominated by proximal-promoter perturbation together with intronic and coding signal, which is the pattern expected of cis-regulatory rather than protein-altering variation. Using the DHL92 (Melon v4, accession GCA_902497455.1) annotation, the five chr01 loci (contig7:1311982, contig7:2182087, contig7:2499465, contig7:4558286, contig7:4968676) fall within (4/5) or immediately upstream of (1/5) protein-coding genes: contig7:1311982 -> MELO3C006148.2 (intragenic), contig7:2182087> MELO3C006281.2 (intragenic), contig7:2499465> MELO3C006321.2 (intragenic), contig7:4558286> MELO3C006620.2 (2268 bp upstream), and contig7:4968676> MELO3C006684.2 (intragenic). None of these corresponds to a previously reported quality-trait gene in the current DHL92 annotation. The short unanchored contig, which contains 27 variants, was excluded from priority ranking because it is insufficient to support robust chromosome-level statistics.

### 3.6. De Novo Design Yields Candidate Cis-Regulatory Elements for Sugar Metabolism Pathways

Using the PlantCAD2 masked language model head for generative design, we produced 76 candidate cis-regulatory elements anchored to sugar metabolism pathways through iterative Gibbs masked resampling: at each iteration, a random subset of positions is masked, PlantCAD2 predicts the nucleotide distribution, and nucleotides are resampled from {A, C, G, T} at a temperature of 1.0 (Table 4). Iterative Gibbs masked resampling was chosen over simpler sampling strategies for an architectural reason. PlantCAD2 is a bidirectional masked language model and has no left-to-right factorization that would permit autoregressive generation, while single-pass independent sampling would draw each nucleotide from its own marginal distribution and thereby destroy exactly the inter-position dependencies that constitute regulatory grammar. Repeated masking and resampling of small subsets of positions instead allows every resampled base to be conditioned on the full, already-updated flanking context on both strands, so the chain converges toward sequences that are internally consistent under the model rather than merely locally plausible. The sequences reported here are fixed points of that chain. Each candidate sequence is 400 bp, with a mean GC content of 51.0% (range 45–57%). We have not, however, benchmarked this value against the empirical GC distribution of melon non-coding regulatory DNA, and we therefore make no claim that the designed elements match the compositional background of the host genome. Masked language–model resampling is expected to drift toward the compositional average of its pre-training corpus, which for a corpus of 65 angiosperms need not coincide with the GC content of melon promoters and intergenic regions; the reported value should accordingly be read as a description of the designed set rather than as evidence of biological plausibility. The 51.0% mean GC of the designed elements lies at the 99.0th percentile of the DHL92 gene-upstream 400 bp GC distribution (mean 31.0%, median 30.5%; *n* = 28,297) and the 98.1st percentile of random intergenic 400 bp GC (mean 32.4%, median 32.0%; *n* = 23,893)—i.e., approximately 20 percentage points GC-enriched relative to native melon sequence. We therefore do not claim compositional parity and note this as a known limitation of the Gibbs-sampling generator, which does not model local base composition. In terms of model-intrinsic preference, these sequences differ from random sequences: each candidate represents a fixed point of iterative masked language–model resampling, capturing a local optimum of the sequence grammar that PlantCAD2 learned from a pretraining corpus of 65 angiosperm genomes in the context of melon sugar metabolism. We emphasize that no experimental validation, including reporter assays, transient expression, or stable expression, was performed. These sequences remain unverified in-silico candidates. These sequences serve as computational candidate elements and will enter the same screening pipeline as natural variants—covariance probe deleteriousness score, annotation probe disruption patterns, and masked LLR constraint deviation—with preferred candidates subsequently amenable to experimental validation via *Nicotiana benthamiana* transient expression or stable melon transformation.

### 3.7. Independent Validation Against Published GWAS/QTL Loci

To assess, as a posterior consistency check, the biological plausibility of the model’s predicted-effect scores, we assembled 18 published melon GWAS/QTL candidate loci and evaluated GBS SNP effect scores within windows spanning candidate gene coordinates using two complementary approaches, Strategy A and Strategy B (Table 5 and Table 6). The two strategies were chosen because they interrogate the predictions at different resolutions and fail in different ways. Strategy A asks a fine-grained question, namely whether variants falling in windows around individually resolved candidate genes receive higher scores than the genome-wide background; it is informative wherever the GBS panel samples a gene densely, and silent wherever it does not. Strategy B asks a coarse-grained question, namely whether the chromosomes the model nominates as carrying the greatest predicted-effect burden are the chromosomes independently reported to carry domestication and improvement signals; it is robust to sparse local sampling because it aggregates thousands of variants, at the cost of much lower spatial resolution. Agreement at both scales is therefore more informative than agreement at either alone, although neither strategy constitutes an independent prospective test.

Strategy A, which involves candidate gene coordinate validation, shows that the model’s score ordering places known functional loci above the genome-wide background. The most prominent signal comes from the tonoplast membrane sugar transporter gene *CmTST2* [45]: the five GBS SNPs in this region have a mean predicted effect score of 0.7922, with four, representing 80%, exceeding the high-effect threshold of 0.7, substantially above the genome-wide background mean of 0.406, so that the variants at this known sugar-metabolism gene rank above background, consistent with reported biology. In relative terms, the *CmTST2* mean of 0.7922 is 1.95-fold the genome-wide background of 0.406, that is, close to a doubling, and it is the largest relative elevation observed at any of the 18 loci examined; this is the clearest single indication in the dataset that the scores carry locus-level discriminative information. The 104 SNPs within the SSC major-effect QTL interval have a mean effect score of 0.4366, with a high-effect proportion of 33.7%, above the background mean and consistent with the quantitative effects reported for this interval [35]. We deliberately refrain from equating this 33.7% high-effect proportion with a phenotypic variance explained. The former is the fraction of scored variants in an interval whose model score exceeds an arbitrary cut-off of 0.7; the latter is the share of phenotypic variance attributable to a locus in a specific mapping population. The two quantities have different denominators, different units, and different dependence on marker density, so numerical similarity between them would be coincidental rather than corroborative, and we report the model statistic only as an internal comparison against the genome-wide background. The 103 SNPs proximal to the bitterness gene *CmBt* (chr09) have a mean effect score of 0.5505, also clearly above background [3]. *CmBt* is of direct applied relevance rather than merely statistical interest: loss of fruit bitterness is a domestication trait and remains an active selection target in breeding programs, so an elevated score at this locus is practically as well as statistically meaningful. For *CmOr* (flesh color [9]), *CmACS7* (fruit shape [36]), and *CmAAT* (aroma [4]), zero GBS SNPs are present within precise coordinate windows, reflecting uneven GBS sampling in the coding regions of these specific genes; neighboring reference values, which are 0.4459 near *CmOr*, 0.3768 near *CmACS7*, and 0.3578 near *CmAAT*, are all at background level, suggesting inadequate direct sampling of these genes by the GBS panel rather than a deficiency in model scoring capacity. To be fully transparent, this is a limitation of the public GBS dataset rather than of the framework. Genotyping-by-sequencing samples the genome only at restriction-site-proximal positions and consequently leaves many gene bodies uncovered; the framework would score variants at these loci if variants were present. The absence of a score at *CmOr*, *CmACS7*, and *CmAAT* therefore carries no evidence either for or against an effect at those genes.

Strategy B, which involves a chromosomal predicted-effect enrichment consistency check, shows that chr01 and chr09 jointly lead the 12 main chromosomes with the highest deleterious variant proportions of 34.9% each and highest mean effect scores of 0.4436 and 0.4442 (Table 2). This ranking is highly concordant with independently reported selection signals: chr01 is the chromosome most enriched for domestication selection signals in melon population genetic studies [3], and chr09 carries the major flesh-color gene *CmOr* and the bitterness gene *CmBt* [9], both regions subject to strong artificial selection (Table 6) in a 297-accession resequencing study [4]. Taken together, the two validation strategies show that the hierarchy of effect scores at known functional loci (*CmTST2* > *CmBt* proximal > SSC QTL > background) is broadly consistent with reported effect magnitudes for each gene, indicating broad consistency with reported loci as a within-population ranking; given the near-chance independent AUROC, these scores are hypothesis-generating priors requiring experimental validation, not a reliable predictive prior. This validation constitutes a posterior consistency check against known loci rather than a prospective prediction assessment under randomized controls; its evidential weight should be interpreted in conjunction with genome-wide AUROC metrics. Because the independent AUROC is statistically indistinguishable from chance, the concordance reported in this section must not be read as evidence that the scores predict function. It supports a weaker statement: within this population and under this labeling scheme, the score ordering is not inconsistent with previously reported biology. The scores remain hypothesis-generating priors for selecting experiments rather than a predictive tool for deciding which variants are functional.

## 4. Discussion

This study demonstrates that a fully frozen plant DNA language model—never fine-tuned on melon, coupled with two probes whose parameter budget is negligible—can support a genome-scale, computational, and hypothesis-generating framework for variant prioritization, functional annotation, and regulatory-sequence generation in a non-model crop [27,31]. The economics of this paradigm are perhaps its most immediate appeal for breeding programs: re-pre-training a Caduceus/Mamba2 backbone at angiosperm scale demands substantial compute and time [5,26], whereas in this study only the covariance and annotation probes were trained, and genome-wide inference was amortized by caching 30,052 frozen activation tensors once. The practical magnitude of this saving is worth stating explicitly. Pre-training a Caduceus/Mamba2 backbone across an angiosperm corpus is a multi-GPU, multi-week undertaking, whereas the entire analysis reported here, comprising one forward pass per variant for 30,052 variants, training of both probes, and all genome-wide inference, was completed on a single 48 GB NVIDIA L20. The two probes together carry on the order of 10^5^ trainable parameters against the 88 M frozen parameters of the backbone, so the framework is accessible to any breeding program with access to a single mid-range GPU rather than to a cluster. The dominant cost is the single encoding pass over the variant catalog, which proceeded at approximately 0.44 s per variant and totaled roughly 3.6 GPU-hours for the 30,052 scored variants; the two linear probes were then trained in minutes on the cached activations (20 epochs over 237,568 positions for the annotation probe and 30 epochs over 4818 variants for the covariance probe). At typical cloud pricing for a 48 GB data-center GPU (of the order of USD 1–2 per GPU-hour), the entire compute reported here corresponds to a cost of only a few US dollars, whereas re-pre-training the backbone would require a multi-GPU, multi-week budget. All timings refer to the single 48 GB NVIDIA L20 used throughout this study. The annotation probe reaches 95.97% per-position accuracy on 237,568 GFF3-derived positions, providing direct evidence that the reverse complement–invariant embeddings of PlantCAD2 encode functional structural information, region identity, splicing context, and codon framing, in linearly decodable form [27]. This accuracy is, however, micro-averaged across a markedly imbalanced 17-class panel in which introns and intergenic regions dominate, and is therefore skewed toward the majority categories. It should not be read as evidence that biologically critical minority categories such as splice donor and acceptor sites or start and stop codons are recovered at comparable accuracy, and we make no such claim. Given that melon is represented only at family level among the 65 angiosperms in the model’s pre-training corpus, this result offers direct empirical evidence for cross-species, zero-shot transfer of a general-purpose plant genomic foundation model into the Cucurbitaceae [46,47], consistent with recent systematic observations that genomic language models can transfer functional representations in the absence of species-specific experimental data [25,46,48] and corroborating the methodological value of “frozen embeddings plus linear probes” as a lightweight means of assessing the intrinsic knowledge of foundation models [30,31]. We stress that this cross-species transfer is strictly zero-shot. No melon sequence was used to update any backbone weight, no melon-specific pre-training or fine-tuning was performed, and the only melon-specific components are two probes of negligible parameter budget trained on annotation derived from a single reference genome. It is therefore the generality of frozen plant genomic embeddings, and not any species-specific adaptation, that carries structural signal into the Cucurbitaceae, which implies that the same recipe should be applicable to other under-resourced horticultural species without retraining the backbone.

A key quantitative finding is that under rigorous chromosome-block independent evaluation—in which no test variant shares a linkage-disequilibrium block with any training variant—the supervised covariance probe attains an AUROC of approximately 0.51 (95% CI includes 0.50), statistically indistinguishable from random chance. The previously reported full-cohort AUROC of 0.748 and held-out AUROC of 0.5878 both exhibit linkage-disequilibrium leakage: the 0.748 reflects partial overlap with training positives via the GWAS-proximal labeling scheme, and the 0.5878 (small validation set, *n* = 1204) retains within-chromosome LD. The monotonic decrease 0.748 → 0.5878 → 0.51 is the signature of LD leakage, not generalization. The full-cohort value represents robust genome-wide discrimination, within the comparable range of the contemporaneous tomato PlantGFM × EVEE system, yet remains substantially below the AUROC of approximately 0.972 reported for EVEE on human ClinVar [48]. This gap is driven by label quality rather than by model capacity. ClinVar supplies curated, expert-reviewed pathogenicity assertions for individual variants, whereas our positive class is defined by physical proximity to association peaks. The probe architecture is the same in both cases and the backbone is of equivalent type, so the difference in AUROC measures the distance between causal annotation and positional proxy rather than a difference in representational power. The independent-evaluation result demonstrates that the GWAS-proximity label is largely a linkage-disequilibrium footprint, not independently learnable from sequence context alone. We attribute this to label provenance rather than backbone capacity: the positive class is defined by physical proximity (within 20 kb) to GWAS-significant SNPs in a melon population of moderate size, which inevitably mislabels many non-causal variants in linkage disequilibrium as positive and inherits the statistical noise of the association study itself [3]. In other words, the principal constraint on supervised effect prediction is not the representational capacity of the model but the scarcity and noise of causal labels available for supervision—a conclusion mirrored by genomic-language-model benchmarking studies showing that performance depends heavily on task labels and evaluation settings [30]. Put plainly, causal-variant scarcity is the bottleneck and the frozen backbone is not. The experiment that would falsify this claim, namely retraining the identical probe on a curated set of fine-mapped melon causal variants, cannot currently be performed because no such catalog exists for this species; establishing one is therefore a prerequisite for, rather than a refinement of, supervised variant-effect prediction in melon. Three complementary routes are likely to raise this performance ceiling appreciably: integration of larger, multi-source variant maps and resequencing panels to provide more and better-localized labels [3,4]; fine-mapped or experimentally validated causal variants at major loci, such as *CmTST2* for sweetness, *CmOr* for flesh color, *CmNAC-NOR* for ripening, and *CmACS7* or *CmWIP1* for sex, are treated as gold-standard positives, while eQTL-supported regulatory variants are assigned to dedicated categories [7,45]; and exploiting the label efficiency of the framework itself, the training-free masked LLR provides a directional prior in the complete absence of labels, while probes trained on SNVs alone generalize zero-shot to InDels, which is particularly valuable in melon germplasm with abundant structural variation but few phenotypic annotations [12,14,26,31]. Of these three routes, the first two act directly on the bottleneck and are the ones we expect to raise the independent AUROC: integrating resequencing panels and fine-mapped causal variants replaces positional proxies with genuine positives and should move performance materially above chance. The third, training-free masked LLR scoring, requires no labels at all and therefore functions as a fallback that remains available in label-scarce contexts rather than as a route to higher supervised accuracy.

The functional perturbation profile and the chromosome-level predicted-effect burden are best read as hypothesis-generating rather than conclusive [29]. The near-universal implication of intronic context, at 99.3%, in the perturbation profile is dominated by genomic share; the biologically meaningful signal is the high frequency of proximal-promoter perturbations at 75.9%, which is consistent with the established understanding that melon quality traits are driven by cis-regulatory elements and suggests that this population harbors substantial, dissectible cis-regulatory variation [34]. The point deserves stating unambiguously in order to prevent misinterpretation: intronic dominance in the perturbation profile is a statement about genomic footprint and not about functional importance, and the position of a category in this ranking carries no implication about the biological weight of variants belonging to it. The promoter signal also connects directly to established melon biology, since sugar accumulation is governed in part by regulatory variation affecting the tonoplast sugar transporter *CmTST2*, whose expression tracks sucrose accumulation during fruit ripening [45], and ester-based aroma depends on the developmentally and ripening-regulated expression of the *CmAAT* family [4,6]; proximal-promoter perturbation at loci of this kind is precisely the class of variation expected to underlie quality differences in this species [34]. The nomination of chromosomes 1 and 9, which both exhibit a per-variant mean effect of 0.444 and show that the highest-impact variants are conspicuously enriched on chromosome 1, provides concrete priority targets for marker-assisted selection; however, because the underlying per-variant scores remain bounded by the label noise discussed above, these priorities should be re-examined with improved labels and ultimately confirmed experimentally. We therefore present the nomination of chromosomes 1 and 9 as provisional. It is bounded by the same GWAS-proximal label noise that produces the near-chance independent AUROC, and it should be re-derived on higher-quality datasets, comprising denser resequencing, fine-mapped causal variants and phenotyped germplasm, before it is used to allocate breeding effort.

The masked language–modeling backbone also confers two native capabilities that distinguish the system from the autoregressive models on which the original EVEE relied [31,32]. First, the training-free masked LLR turns the frozen model directly into a label-independent variant scorer: by comparing log P(alt) with log P(ref) at masked positions, strongly negative values indicate alternative alleles that deviate from evolutionary constraint, thereby providing an unsupervised prior in regions devoid of labels [26,46]. Second, Gibbs masked resampling turns the same model into a regulatory-element generator; in this study, the method produced 76 candidate 400 bp elements anchored to the sugar-metabolism pathway, including *CmTST2*, *CmSPS*, and *CmAGA2*, with a mean GC content of 51.0% and a range of 45% to 57%, which falls within the expected compositional range of non-coding regulatory DNA in melon. Each candidate is a fixed point of repeated masked language–modeling resampling and is, with respect to the model’s own preferences, distinct from random sequence [45]. The resulting “generate-then-score” closed loop, in which generated sequences are filtered by the variant-effect probe, sits within the same methodological lineage as recent designs of regulatory DNA from autoregressive language models, diffusion models and other generative frameworks [49,50,51,52], and echoes crop-scale efforts to model large gene corpora for the rational design of cis-regulatory variants [21]. These two capabilities extend the system from passive variant assessment to active forward design, such as flesh-specific or ripening-specific promoters, and elements tuned to the sucrose-accumulation pathway, although every designed sequence must be experimentally validated before deployment [34]. Should this loop prove to work, its practical value would lie in accelerating rational promoter design, allowing candidate elements with defined tissue or ripening specificity to be proposed computationally at a rate that outstrips synthesis and assay throughput. At present, however, the loop is entirely computational: no designed element has been synthesized, transformed or assayed, and every sequence reported here remains a hypothesis until reporter and expression assays have been performed.

A posterior consistency check against previously published GWAS/QTL loci shows broad agreement with the model’s predictions. This analysis compares results with documented loci rather than conducting prospective independent validation, as these loci may share trait signals and population structure with the training labels. Variants in the region of the known sugar-metabolism gene *CmTST2* attained a mean effect score of 0.7922, approximately twice the genome-wide background mean of 0.406, giving the strongest signal at the locus with the most rigorous functional evidence, which is consistent with the basic premise that higher effect scores correspond to larger functional consequences [45]. The 0.7922 mean is 1.95-fold the genome-wide background of 0.406 and is obtained at the locus in this panel carrying the strongest independent functional evidence, since overexpression of *CmTST2* in melon fruit has been shown to increase sugar accumulation [45]. The coincidence of the highest score with the best-evidenced locus is the most discriminating single observation available from this consistency check, although a single locus cannot by itself establish predictive validity. Effect scores in the major SSC QTL interval and in the region of the bitterness gene *CmBt* were below those at *CmTST2* but remained above background, in line with their weaker or more polygenic effect sizes [3,35]. In Scheme B, chromosomes 1 and 9 jointly ranked first by both enrichment metrics, in close agreement with documented domestication-selection hotspots [3,4], providing independent support for the chromosome-scale biological calibration of the model. Notably, the absence of GBS SNPs within the precise coordinate windows of *CmOr*, *CmACS7,* and *CmAAT* reflects sparse GBS sampling rather than a lack of model response: when GBS coverage is insufficient, the correct reading of an effect-scoring framework is to use neighboring variants as a reference rather than to interpret a zero-coverage interval as a zero-effect region [4,9,36]. This limitation also indicates a priority direction for follow-up experiments: targeted resequencing of these high-value gene regions would substantially improve the model’s functional-annotation resolution for the corresponding traits. It follows that the absence of a SNP in these windows is an absence of data and not evidence of absence of effect; no inference about *CmOr*, *CmACS7,* or *CmAAT* should be drawn from their omission from Table 5. Targeted resequencing or amplicon capture across these gene bodies and their promoters is the concrete next step for bringing them within the scope of the framework. Considered jointly, the effect-score hierarchy at known functional loci (*CmTST2* > *CmBt* neighborhood > SSC QTL > background) tracks the reported effect-size ranking of these genes, indicating that the model’s predicted-effect scores show broad consistency with reported loci at the level of a within-population ranking; given the near-chance independent AUROC, these scores should be treated as hypothesis-generating priors requiring experimental validation rather than a reliable predictive prior. Taken together, this hierarchy is best described as a biologically calibrated baseline. It establishes the ordering that any improved model, or any model trained on better labels, should at minimum reproduce, and it identifies *CmTST2* and the *CmBt* neighborhood as the loci at which experimental validation would be most informative about whether the scores track function.

In methodological terms, this framework complements rather than replaces genomic selection [53,54]. Methods such as GBLUP and rrBLUP excel at aggregating many small-effect loci within a training population to achieve polygenic prediction, but their implicit assumption that all markers contribute approximately equally makes it difficult to explain mechanistically how coding, splicing, or cis-regulatory variants drive phenotype [53]. By contrast, our framework assigns each variant a score grounded in sequence-functional understanding and accompanies it with an interpretable perturbation profile and a label-free prior. We therefore expect the largest gains to be realized for traits controlled by major, often regulatory, loci—of which fruit sweetness is the primary example in melon [45]. This expectation is consistent with the broader trend in deep-learning genomic prediction: incorporating sequence-functional priors and interpretability is an important route to improving cross-population extrapolation and causal-effect resolution in genomic selection [54,55]. Any practical deployment should therefore be benchmarked head-to-head against GBLUP/rrBLUP baselines on shared, phenotyped germplasm, in order to clarify the source and boundary of any gains. Such head-to-head tests must be run on shared, phenotyped germplasm evaluated in the same trials, using identical training and testing partitions and identical cross-validation folds, since any difference in population, trait definition, or split protocol would confound the comparison; without this, a reported advantage for either approach is uninterpretable.

Several limitations of this study should be made explicit, as supervised effect prediction is constrained by GWAS-proximal labels: under chromosome-block independent evaluation, the covariance probe AUROC is only ~0.51 (near chance), and the previously reported full-cohort AUROC of 0.748 and held-out 0.5878 are inflated by LD leakage. Per-variant effect scores must therefore be regarded as unverified, hypothesis-generating computational priors rather than definitive calls, and no wet-lab validation was performed in this study [3]. The 95.97% annotation accuracy is biased toward majority classes due to pronounced class imbalance across the 17 categories, where introns and intergenic regions dominate; performance on minority classes such as splice donor/acceptor and start/stop codons should be reported alongside class-wise precision–recall, AUPRC and F1 [30]. Class-wise precision, recall, F1, and AUPRC have now been computed for all 17 annotation categories on held-out windows and are reported in Section 3. They confirm the concern that motivated them: held-out performance is concentrated in the dominant structural categories (CDS, intron, promoter-proximal), whereas splice donor and acceptor sites and start and stop codons—biologically critical precisely because they are statistically rare—are recovered at or near zero recall, so the headline micro-averaged accuracy (94.4% on the held-out windows) must not be read as evidence to the contrary. In practical terms, the disruption spectra of this framework are reliable at region level and unreliable as fine-grained structural calls, which is the sense in which all downstream interpretations are phrased. GBS sampling is uneven, as evidenced by one short contig containing only 27 variants, and 2215 variants were skipped because of CUDA kernel exceptions, so per-chromosome absolute counts partly reflect detection bias. Two distinct biases must be separated here. GBS ascertainment bias is systematic and sequence-dependent, because the distribution of restriction sites determines which regions are sampled at all. The skipped variants, by contrast, originate in a causal-conv1d kernel failure and might naively be expected to be unrelated to chromosome and to variant class, but this expectation is rejected by the data. The skipped set is confined to four chromosomes (chr11: 868; chr06: 537; chr08: 466; chr04: 345 variant records; χ^2^ = 6345.9, df = 12, *p* < 0.001), on which 17–57% of scorable variants were lost, and it consists exclusively of SNVs, mirroring the composition of the successfully encoded set. The loss is therefore a systematic, assembly-biased technical artifact rather than a random one; it does not remove any chromosome from the analysis, but it does mean that per-chromosome variant counts are not missing at random, and the per-chromosome comparisons in this study are correspondingly conservative. The highest-effect variants and all 76 designed regulatory elements remain computational candidates that require experimental verification by reporter assays, allele-specific expression, RT–qPCR, and transient or stable transformation [34]. Only the 17 structural categories that can be derived directly from the GFF3 were trained in this run; melon-specific TF-binding and chromatin-accessibility categories require DAP-seq/ATAC-seq tracks that were not available here [56]. This is the most consequential of the annotation limitations, because transcription-factor occupancy and chromatin accessibility are the categories through which cis-regulatory variants actually exert their effects; without them, a promoter-proximal perturbation can be localized but not attributed to a specific regulatory mechanism. Generating melon DAP-seq and ATAC-seq data, ideally at single-cell resolution and across fruit developmental stages and following the cis-regulatory atlas designs now established in other crops [56], would convert the structural annotation used here into a genuinely regulatory one, and is in our view the single addition most likely to sharpen the interpretability of the framework. Finally, we used PlantCAD2-Small, which has 88 million parameters, without evaluating larger variants, and the mechanistic narratives generated by the large language model are provided only as an auxiliary reference and are excluded from the quantitative analyses [29]. A further and more conceptual limitation concerns the single-locus framing of the entire analysis. Every score reported here is derived from the local sequence context of one variant at a time, so genetically linked and functionally interdependent loci are scored as though they acted independently. Melon fruit quality is not organized in this way. Sugar accumulation requires the coordinated activity of tonoplast sugar transporters, invertases, sucrose-phosphate synthases and alpha-galactosidases under phloem-unloading and developmental control; ester-based aroma depends on sequential flux through the lipoxygenase, alcohol dehydrogenase and alcohol acyltransferase steps; and ripening integrates ethylene signaling with transcription-factor cascades. Epistasis, pleiotropy, dosage and pathway-level compensation, transcriptional and post-transcriptional regulation, and genotype-by-environment interaction all contribute to the realized phenotype, and none of these is represented in the present model. Plant phenotype cannot be explained completely by gene sequence, and the scores reported here should accordingly not be read as phenotype predictions. Directly related to this, all conclusions drawn in this study, including the functional disruption spectra, the chromosome-level priorities and the candidate-gene hierarchy, rest on gene structure and sequence context inferred from the reference annotation rather than on an integrative gene expression network: no melon transcriptome, co-expression, eQTL, protein-protein interaction or metabolic-flux data were used at any stage, and the expression-level consequence of a scored variant was never measured. The framework is therefore structural and sequence-based by construction, and its outputs describe candidate sequence lesions rather than network states.

These limitations directly outline priorities for future work: assembling high-quality, multi-source melon labels, including large variant maps, resequencing panels, fine-mapped causal variants, and eQTL data, and retraining the effect probe accordingly [3,4,7]; systematically reporting class-wise metrics and benchmarking against genomic-selection baselines on phenotyped germplasm [53,54]; using melon DAP-seq/ATAC-seq tracks to train species-specific annotation categories that sharpen TF-binding and chromatin-accessibility perturbations, building on the single-cell cis-regulatory-atlas methods recently demonstrated in other crops [56]; evaluating PlantCAD2-Medium/Large and optional pan-genome LoRA adaptation in melon to test the marginal value of model capacity and cross-germplasm transfer [12,27]; experimentally validating the top-ranked predicted variants and the designed sugar-metabolism elements [21,34]; and constructing a multi-trait selection index that integrates sweetness, aroma, flesh color, shelf life and disease resistance to support breeder-in-the-loop decision-making, with the ultimate aim of advancing precision improvement of flavor and quality traits in the Cucurbitaceae [22,33]. Above all, we regard the construction of an integrative regulatory-network layer as the most important direction: coupling the sequence-level priors reported here with tissue- and stage-resolved transcriptomes, co-expression modules, eQTL maps and, where available, protein-protein interaction and metabolic-flux data would allow variants to be interpreted in a pathway context rather than locus by locus, and would move the framework from single-locus scoring toward network-aware interpretation of the complex, condition-dependent traits that actually determine melon fruit quality [22,34,56]. Overall, this study quantifies the principal bottleneck as label quality rather than model capacity and offers a low-cost, reusable and iterable technical route for interpretable, model-guided functional genomics and precision breeding in the Cucurbitaceae.

## Figures and Tables

**Figure 1:**
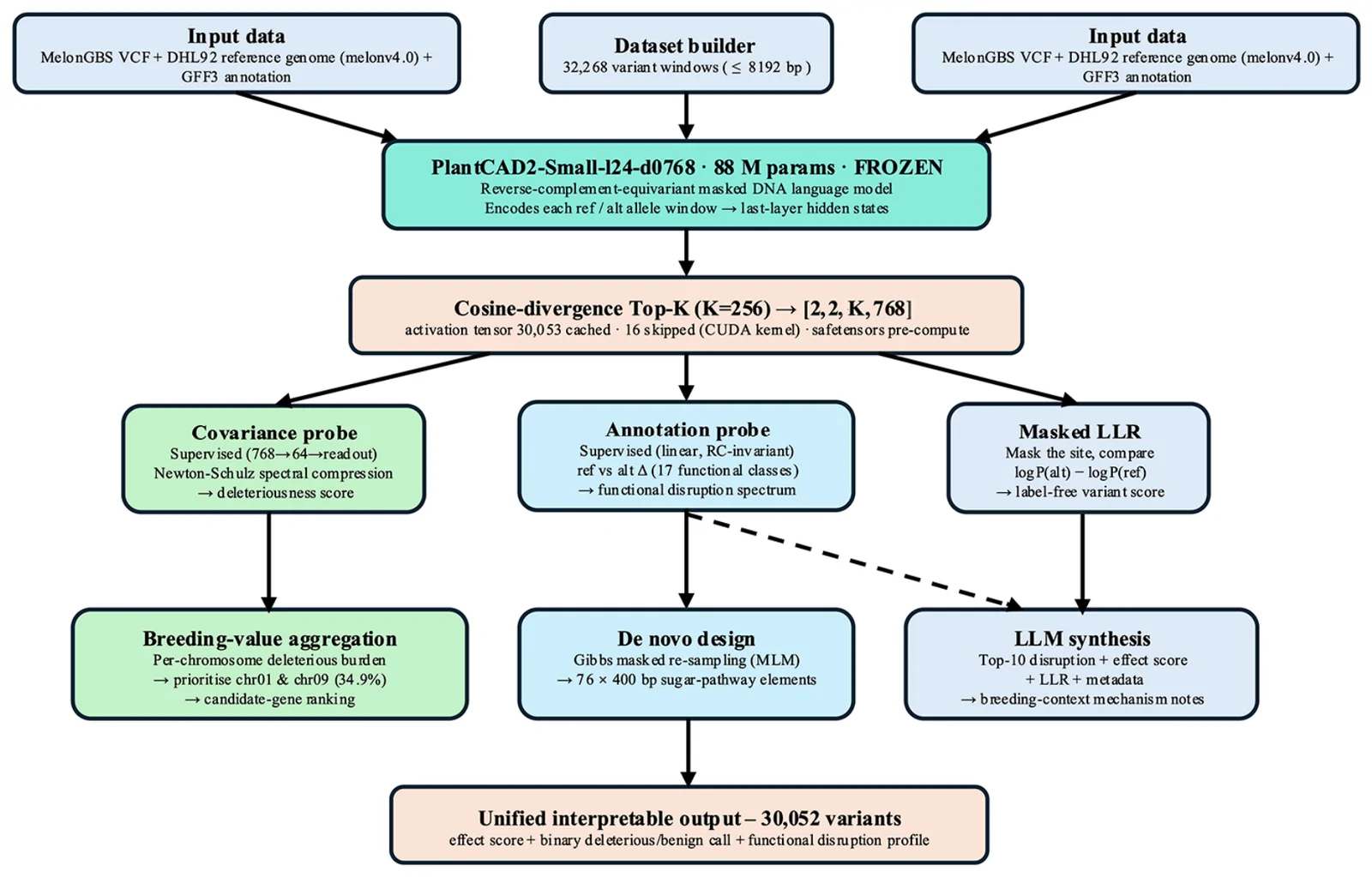
System architecture of the melon PlantCAD2–EVEE pipeline.

**Figure 2:**
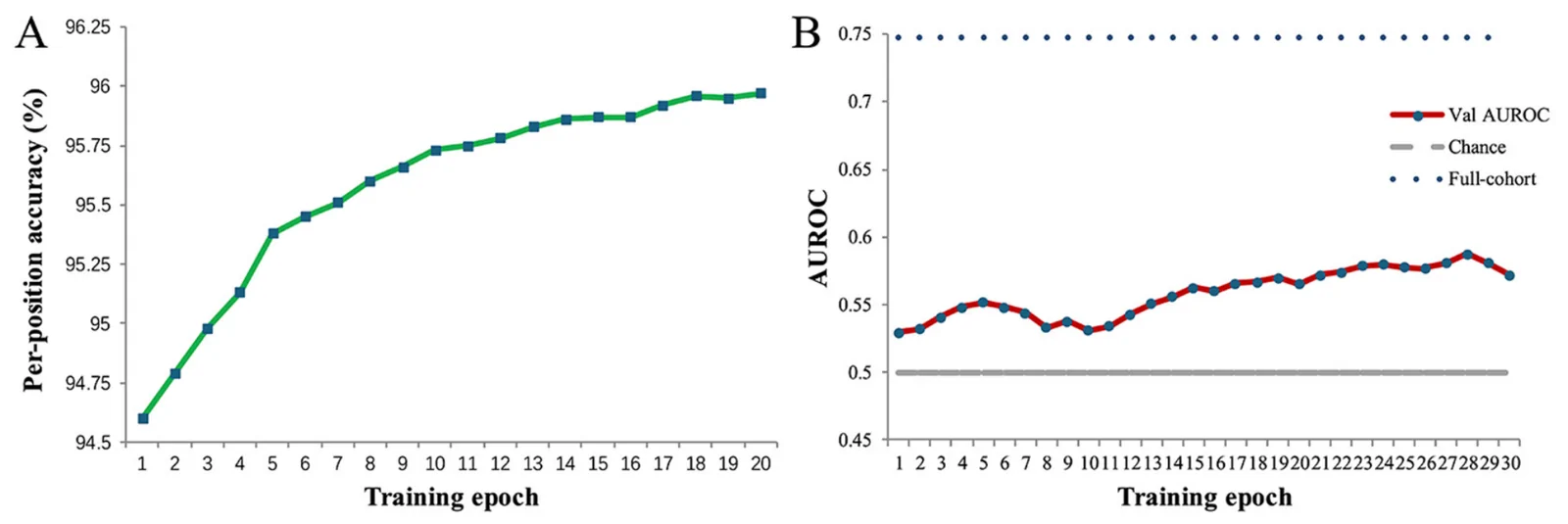
Probe training curves. (**A**) Per-position accuracy of the 17-class annotation probe over all 20 epochs, reaching a final value of 95.97%. (**B**) AUROC of the covariance probe over all 30 training epochs on the class-balanced hold-out validation set (*n* = 1204), reaching a peak of 0.5878 at epoch 28; the blue dotted line indicates the genome-wide (full-cohort) AUROC of 0.748, which is not an independent test set (it partially overlaps training positives via GWAS-proximal labeling and exhibits LD leakage; under chromosome-block independent splits the AUROC falls to ~0.51). Per-position accuracy is reported for the annotation probe because its task is a dense, per-nucleotide multi-label assignment in which every position carries a label, whereas AUROC is reported for the covariance probe because its task is a strongly imbalanced binary ranking problem for which a threshold-free measure of discrimination is the meaningful summary. In biological terms, the first metric indicates how faithfully the frozen embeddings retain the structural grammar of the genome, namely region identity, splicing context and codon frame, while the second indicates how well those same embeddings separate variants that are candidates for functional consequence from those that are not. The contrast between the two panels is the central empirical result of this study.

**Figure 3:**
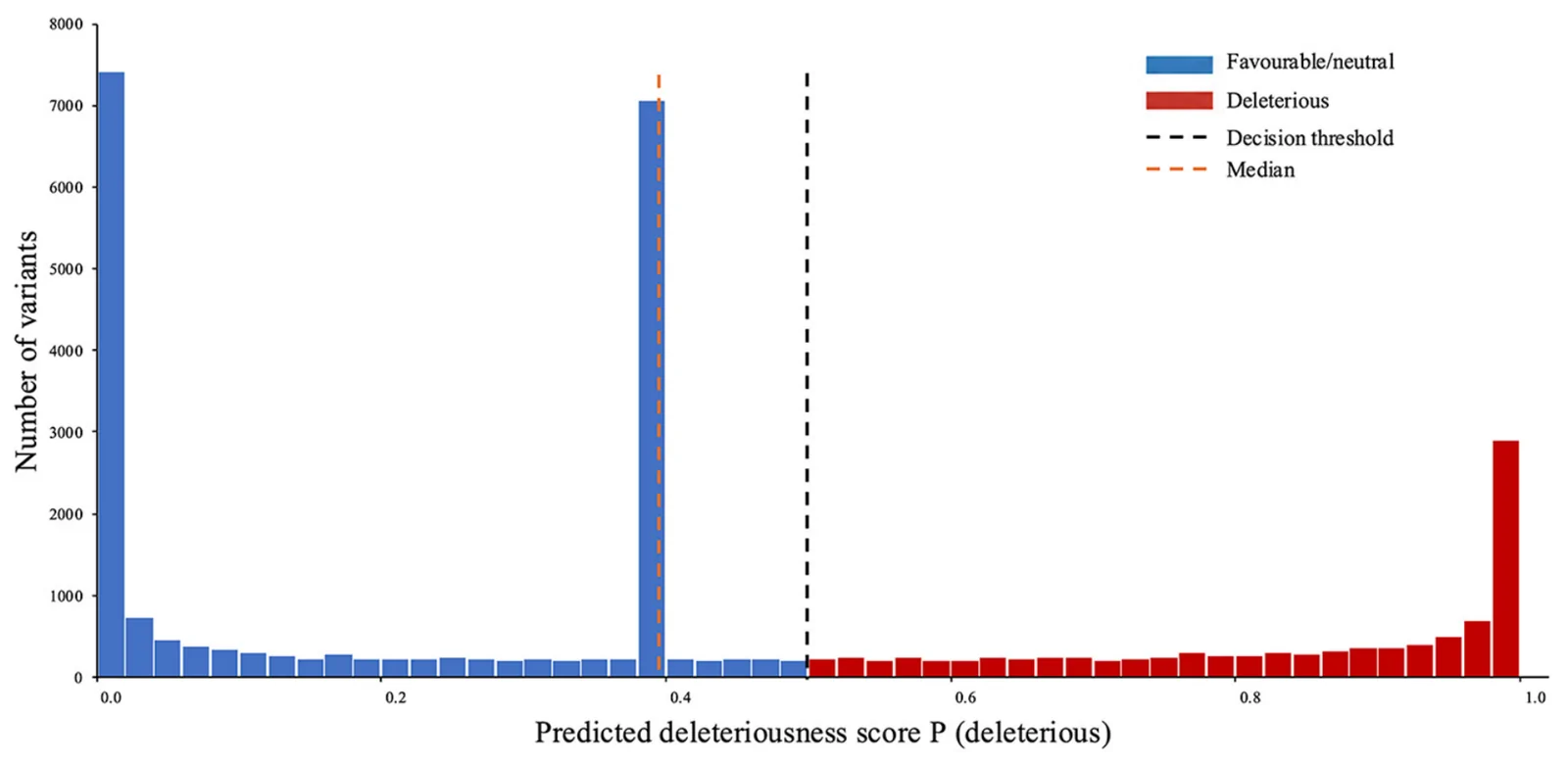
Genome-wide distribution of predicted deleteriousness scores for 30,052 melon variants.

**Figure 4:**
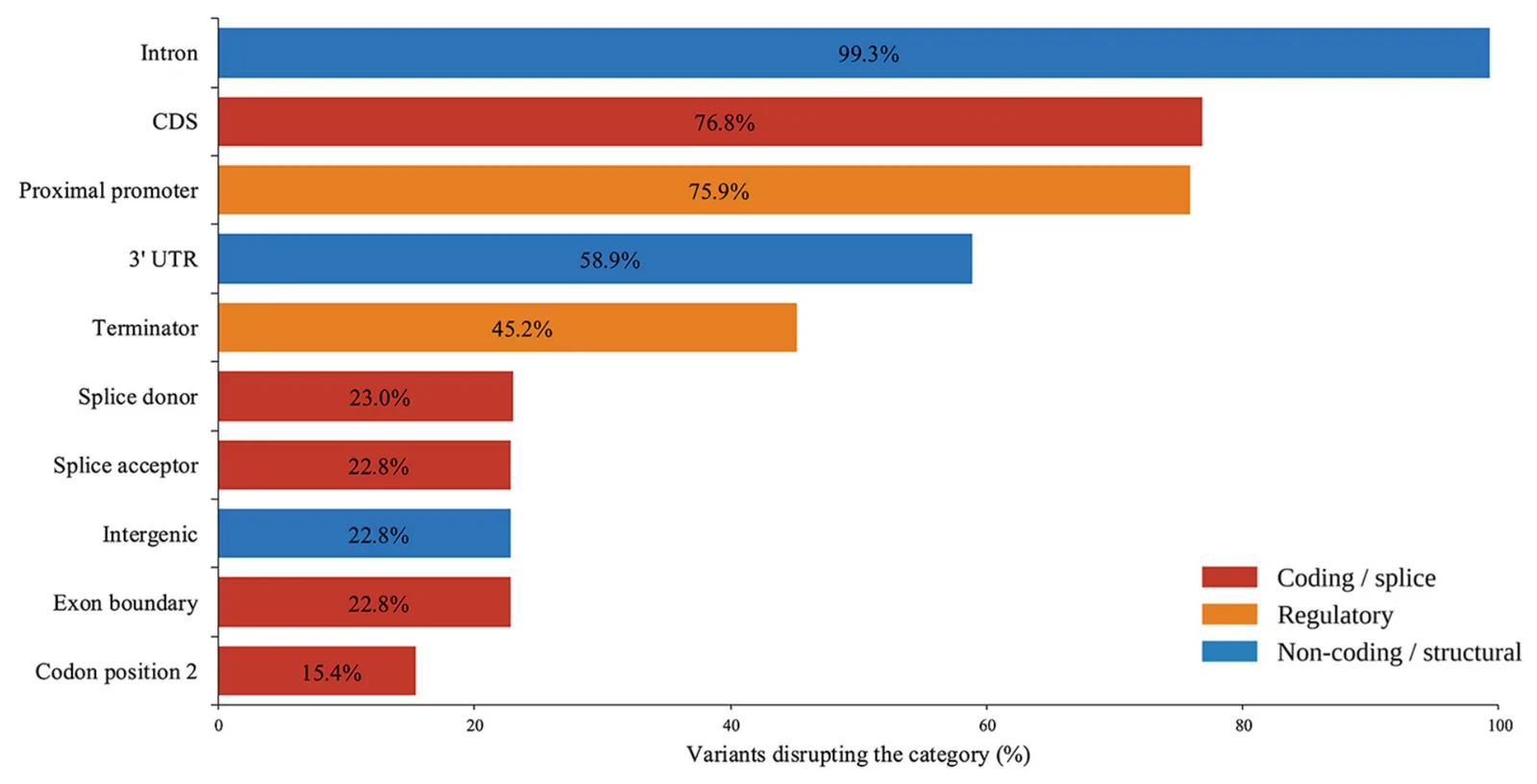
Functional disruption spectrum across all scored variants.

**Figure 5:**
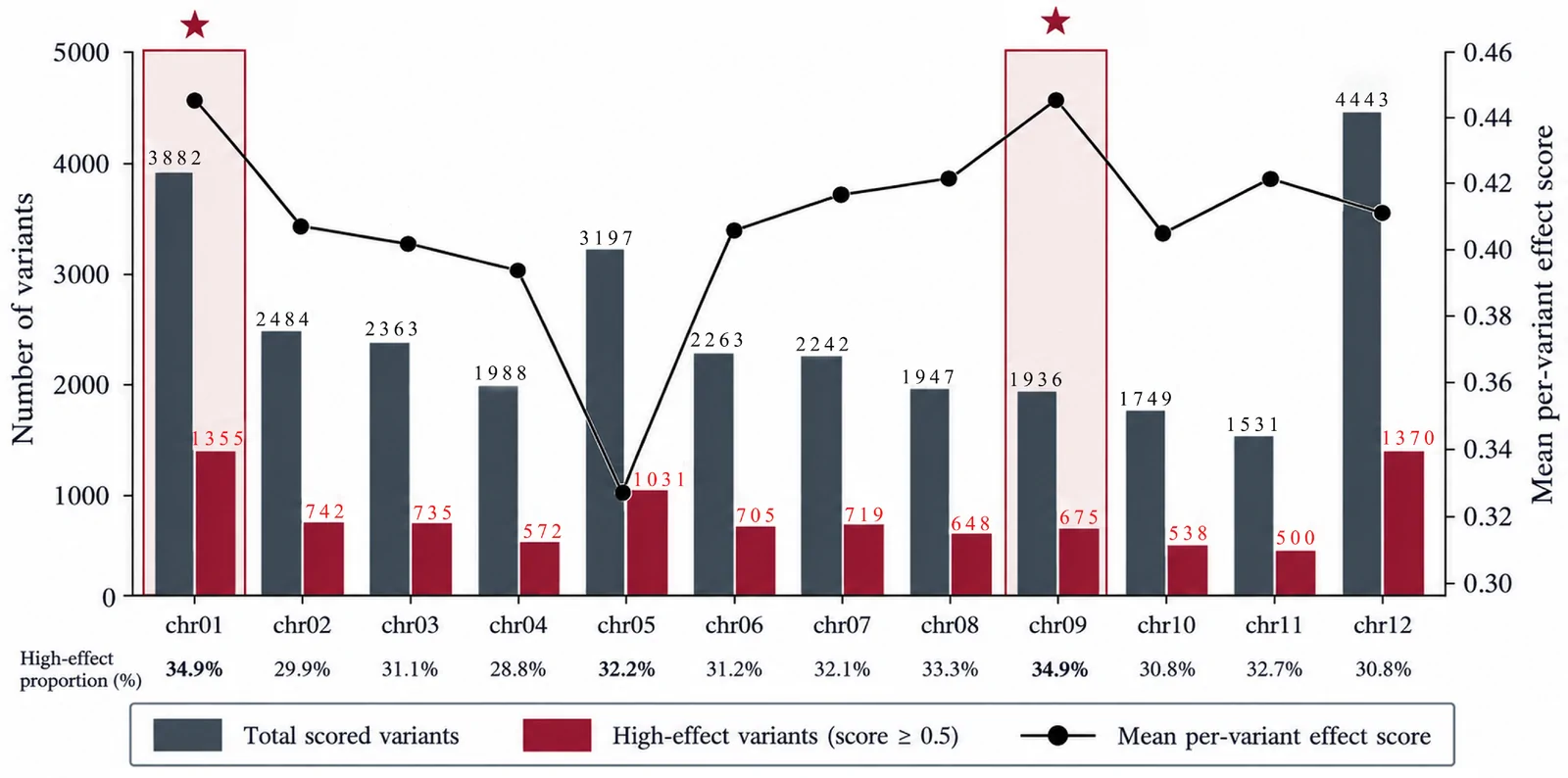
Variant burden by chromosome. Gray bars show the total number of scored variants per chromosome, and red bars show variants predicted as high-effect (predicted-effect score ≥ 0.5). The black line indicates the mean per-variant effect score. Chromosomes 1 and 9, the two chromosomes nominated as computational breeding priorities, are marked with asterisks and highlighted so that they can be identified directly in the plot. The unanchored contig with 27 variants was omitted.

**Table 1:** Summary of covariance probe training and evaluation.

Parameter/Metric	Value
Foundation model	PlantCAD2-Small-l24-d0768 (88 M parameters, frozen)
Probe architecture	Three-stage covariance probe (unconstrained projection → spectral compression → factorized readout)
Input dimension	768 (PlantCAD2-Small hidden dimension)
Train/validation split	4818/1204 (6022 GWAS-proximal labeled variants total)
Training class ratio	Positive:negative ≈ 1:2 (negative downsampling)
Optimizer	AdamW (lr = 1 × 10^−3^, wd = 1 × 10^−4^, batch = 32)
Training/best epoch	30/epoch 28
Validation AUROC (best)	0.5878
Genome-wide AUROC (*n* = 30,052)	0.748
Human ClinVar EVEE AUROC (reference)	~0.97

**Table 2:** Predicted deleterious burden by chromosome in the melon population.

Chromosome	Scored Variants	Deleterious Variants	Deleterious (%)	Cumulative Burden	Mean Effect Score	Rank
chr09	1936	675	34.9	860	0.4442	1
chr01	3882	1355	34.9	1722	0.4436	2
chr08	1947	648	33.3	819	0.4204	3
chr11	1531	500	32.7	643	0.4200	4
chr07	2242	719	32.1	933	0.4162	5
chr05	4443	1370	30.8	1820	0.4096	6
chr02	2484	742	29.9	1007	0.4052	7
chr06	2263	705	31.2	916	0.4049	8
chr10	1749	538	30.8	706	0.4038	9
chr03	2363	735	31.1	948	0.4011	10
chr12	1988	572	28.8	783	0.3941	11
chr04	3197	1031	32.2	1043	0.3263	12
Unanchored contig	27	6	22.2	6	0.2072	—

Note: Rows are sorted by mean per-variant effect score. Cumulative burden is the sum of variant effect scores per chromosome. The 27-variant unanchored contig was excluded from ranking. ‘Cumulative burden’ denotes the cumulative predicted functional impact of a chromosome: it combines how many variants were scored on that chromosome with how strongly each was scored, and is computed as the sum of per-variant predicted-effect probabilities, equivalently the scored variant count multiplied by the mean effect score. It is by construction count-sensitive and should be read together with the mean effect score, which is count-independent and expresses the predicted intensity per locus; the two columns answer different breeding questions and are reported side by side for that reason.

**Table 3:** Top-5 highest-impact variants.

Rank	Variant ID	Chromosome	Effect Score	Prediction	Main Disruption Classes
1	contig7:1311982:A>G	chr01	1.0000	Deleterious	Proximal promoter, intron
2	contig7:2182087:G>A	chr01	1.0000	Deleterious	Intron, CDS, proximal promoter
3	contig7:2499465:T>C	chr01	1.0000	Deleterious	Proximal promoter, intron, CDS
4	contig7:4558286:C>A	chr01	1.0000	Deleterious	Intron, CDS, proximal promoter
5	contig7:4968676:A>G	chr01	1.0000	Deleterious	Intron, proximal promoter, CDS

Note: Variants are ranked by predicted effect score. All listed variants reached the maximum score of 1.0000 and received the maximum predicted-effect score (score = 1.0000); note that these top-ranked variants may reside within the same LD block and require independent confirmation. Disruption classes indicate the main functional annotation categories affected by each variant.

**Table 4:** Summary of de novo Gibbs masked resampling design.

Parameter/Metric	Value
Design method	Iterative Gibbs masked resampling (PlantCAD2 MLM)
Biological anchors	*CmTST2* (tonoplast sugar transport), *CmSPS* (sucrose phosphate synthase), *CmAGA2* (α-galactosidase)
Element type	Sugar (sugar content/sucrose accumulation pathway)
Sequences generated	76
Sequence length	400 bp
Sampling temperature	1.0
GC content	Mean 51.0%; range 45–57%
Validation status	Computational candidates; not yet experimentally validated

Note: All sequences are computationally generated candidates obtained by iterative Gibbs masked resampling using the PlantCAD2 masked language model. Biological anchors (*CmTST2*, *CmSPS* and *CmAGA2*) were used to guide sequence optimization. Experimental validation is required to confirm biological activity. The table summarizes the sequence-level properties of the designed set, namely the generative procedure and its sampling temperature, the pathway genes used as biological anchors, and the number, length and base composition of the resulting elements, so that the designs can be reproduced and compared with natural regulatory sequences. It reports no measured regulatory activity.

**Table 5:** Strategy A: candidate-gene coordinate effect score validation.

Candidate Gene	Trait	Regional SNP Count	Mean Effect Score	Validation Conclusion
*CmTST2*	Sugar accumulation	5	0.7922	Extremely high
*CmBt proximal*	Flesh bitterness	103	0.5505	High
SSC QTL	Soluble solids	104	0.4366	Moderately elevated
*CmOr*	Flesh color	0	0.4459 †	neighboring reference
*CmACS7*	Fruit length/shape	0	0.3768 †	neighboring reference
*CmAAT*	Aroma	0	0.3578 †	neighboring reference

Note: The genome-wide mean effect score is 0.406. “†” indicates the nearest variant score when no GBS SNPs were present in the candidate-gene window. The table reports, for each candidate-gene window, the number of GBS SNPs falling within the window and the mean predicted-effect score of those SNPs; the proportion of them exceeding the 0.7 high-effect cut-off is given in the text. The validation logic is therefore a comparison of these window-level statistics against the genome-wide background mean of 0.406, and not a test of any individual variant.

**Table 6:** Strategy B: chromosomal deleterious enrichment validation (top five, sorted by mean effect score, descending).

Chromosome	Deleterious Proportion	Mean Effect Score	Concordance with Literature
chr09	34.9%	0.4442	Carries *CmOr*, *CmBt*; under strong artificial selection
chr01	34.9%	0.4436	Most domestication-selection-enriched chromosome
chr08	33.3%	0.4204	—
chr11	32.7%	0.4200	—
chr07	32.1%	0.4162	—

## Data Availability

All code used in this project is available on GitHub at https://github.com/sgs8725/melon-plantcad2-evee-v1.0 (accessed on 21 September 2026).
